# *In Vitro* Detection of prionemia in TSE-Infected Cervids and Hamsters

**DOI:** 10.1371/journal.pone.0080203

**Published:** 2013-11-01

**Authors:** Alan M. Elder, Davin M. Henderson, Amy V. Nalls, Jason M. Wilham, Byron W. Caughey, Edward A. Hoover, Anthony E. Kincaid, Jason C. Bartz, Candace K. Mathiason

**Affiliations:** 1 Department of Microbiology, Immunology and Pathology, Colorado State University, Fort Collins, Colorado, United States of America; 2 Rocky Mountain Laboratories, National Institute for Allergy and Infectious Diseases, National Institutes of Health, Hamilton, Montana, United States of America; 3 Medical Microbiology and Immunology, Creighton University, Omaha, Nebraska, United States of America; USGS National Wildlife Health Center, United States of America

## Abstract

Blood-borne transmission of infectious prions during the symptomatic and asymptomatic stages of disease occurs for both human and animal transmissible spongiform encephalopathies (TSEs). The geographical distribution of the cervid TSE, chronic wasting disease (CWD), continues to spread across North America and the prospective number of individuals harboring an asymptomatic infection of human variant Creutzfeldt-Jakob Disease (vCJD) in the United Kingdom has been projected to be ~1 in 3000 residents. Thus, it is important to monitor cervid and human blood products to ensure herd health and human safety. Current methods for detecting blood-associated prions rely primarily upon bioassay in laboratory animals. While bioassay provides high sensitivity and specificity, it requires many months, animals, and it is costly. Here we report modification of the real time quaking-induced conversion (RT-QuIC) assay to detect blood-borne prions in whole blood from prion-infected preclinical white-tailed deer, muntjac deer, and Syrian hamsters, attaining sensitivity of >90% while maintaining 100% specificity. Our results indicate that RT-QuIC methodology as modified can provide consistent and reliable detection of blood-borne prions in preclinical and symptomatic stages of two animal TSEs, offering promise for prionemia detection in other species, including humans.

## Introduction

The hematogenous spread of prions in transmissible spongiform encephalopathy (TSE)-infected animals has long been hypothesized [1-3], but evidence for the presence of prions in non-nervous/lymphoid tissues and blood was not available for several decades [4-8]. Later studies have provided unequivocal proof of efficient TSE blood-borne infectivity [9-14]. The knowledge that prions traffic throughout the body in blood has important implications for both human and animal health.

Variant Creutzfeldt-Jakob disease (vCJD) emerged following the bovine spongiform encephalopathy (BSE) epidemic in the United Kingdom in the 1980s and 90s. Biochemical and strain typing analysis have provided evidence indicating that vCJD originated from human exposure to BSE contaminated material. To date, 227 cases of vCJD have been diagnosed worldwide [15], four of which have been transmitted by non-leukodepleted blood transfusion [16-20]. While leukocyte reduction has been implemented to filter prions and prion carrying cells from blood products, these filtration methods are unable to remove 100% of TSE infectivity [8,21,22]. In addition, recent reports have revealed that 1/1,250 to 1/3,500 persons in the United Kingdom may be asymptomatic carriers of vCJD as a result of the BSE epidemic [23]. Thus, concern exists that a secondary outbreak of vCJD may ensue involving blood-borne prion transmission originating from individuals unknowingly carrying a subclinical prion infection. Here we address the need for an *in vitro* assay with the ability to detect the prion disease-associated isoform of prion protein (PrP^D^) present in whole blood.

Several animal TSEs, including chronic wasting disease (CWD) of deer and elk [13,24] and hamster-adapted transmissible mink encephalopathy (TME) [25,26] exhibit a hematogenous phase of infection, thus providing excellent TSE models for the development of an ante-mortem blood-borne PrP^D^ detection assay.

While traditional assays, such as Western blot and immunohistochemistry (IHC), are effective for detecting large quantities of prions present in nervous and lymphoid tissues, they do not have the ability to detect the minute quantities of prions thought to be present in bodily fluids or peripheral tissues early in infection. Rodent bioassays have the necessary sensitivity and specificity to detect hematogenous prions, but they are not realistic as rapid and cost-effective diagnostic tools. *In vitro* prion detection was advanced with the advent of serial protein misfolding cyclic amplification (sPMCA) [25,27]. sPMCA has been optimized for the detection of prions in blood [26] and requires less time than bioassay, but its use has been hampered by a lack of consistent sensitivity and a dependence on protease digestion prior to immunoassay readout. In contrast, the real-time quaking-induced conversion (RT-QuIC) assay [28-30] relies upon the seeded conversion of recombinant prion protein (rPrP) to PrP^D^ and subsequent binding of the fluorescence marker, thioflavin T (ThT), to the resulting amyloid isoforms [31]. This process offers enhanced ante-mortem prion detection and real-time fluorescence readout [30].

We undertook this project to determine if adaptations applied to RT-QuIC could provide a fast, sensitive and consistent assay for the detection of blood-borne prions.

## Results

### RT-QuIC analysis of whole blood collected in various anticoagulants

**Figure 1:**
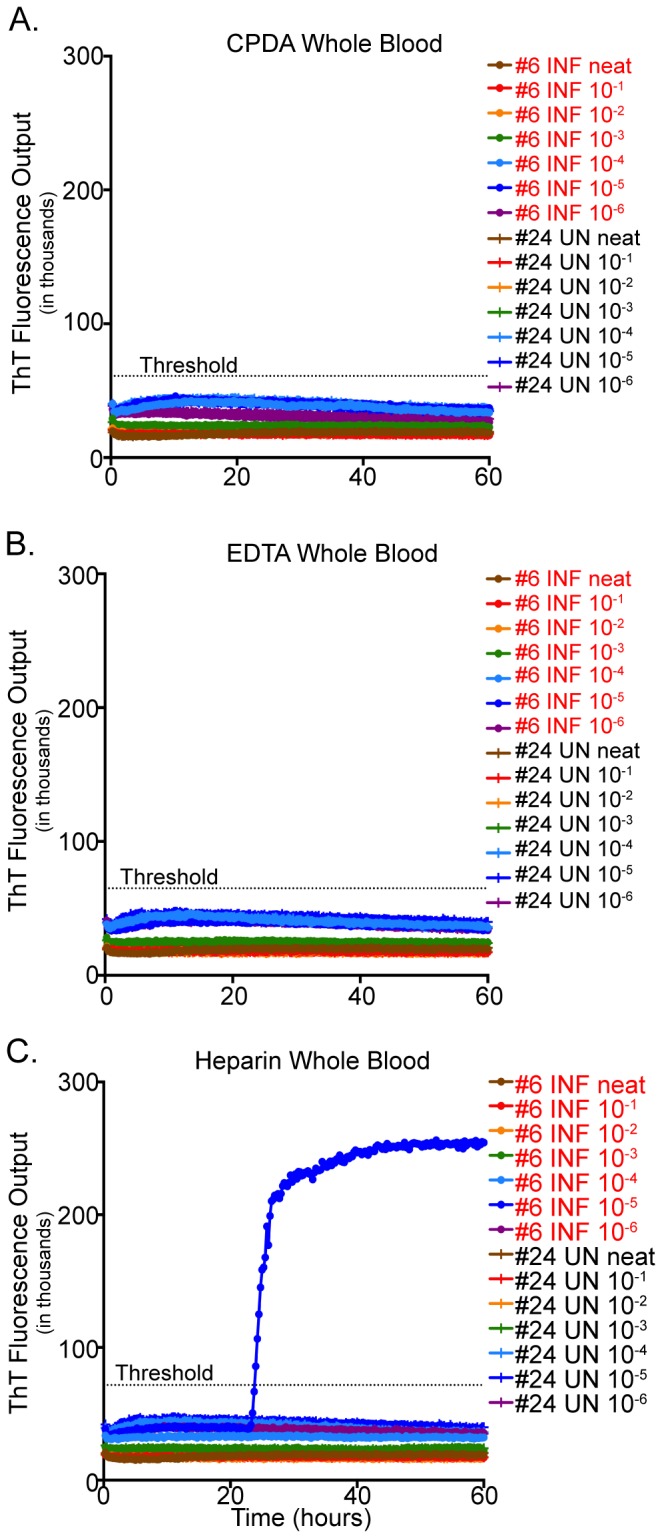
RT-QuIC analysis of whole blood collected in various anticoagulants. Blood was collected from a CWD-infected and CWD-naïve white-tailed deer and preserved in one of three anticoagulants: CPDA, EDTA, or heparin. Serial blood sample dilutions (neat to 10^-6^) were assayed by RT-QuIC for 60 hours and ThT fluorescence level above threshold determined positivity. Detection of PrP^C^-converting activity for each replicate is shown for blood collected in CPDA (A), EDTA (B), and heparin (C).

To determine the influence of common blood preservation reagents in *in vitro* PrP^D^ detection assays, we compared the ability of RT-QuIC to amplify CWD prions in fresh cervid whole blood preserved in CPDA (citrate phosphate dextrose adenine), EDTA (ethylenediaminetetraacetic acid), or heparin. Samples were run in serial dilutions (10^0^-10^-6^) in the RT-QuIC assay to determine the optimal dilution for PrP^D^ detection. While RT-QuIC PrP^C^-converting activity was observed in heparin-preserved blood from CWD-infected deer (1/2 replicates in one dilution; 10^-5^), PrP^C^-converting activity was not detected in CPDA or EDTA preserved blood from the same animal or any blood collected from sham-inoculated deer (Figure 1). This experiment was repeated six times with fresh whole blood with similar results witnessed each time. All subsequent RT-QuIC analyses were conducted on whole blood harvested in heparin. 

### RT-QuIC analysis of fresh versus frozen whole blood

**Figure 2:**
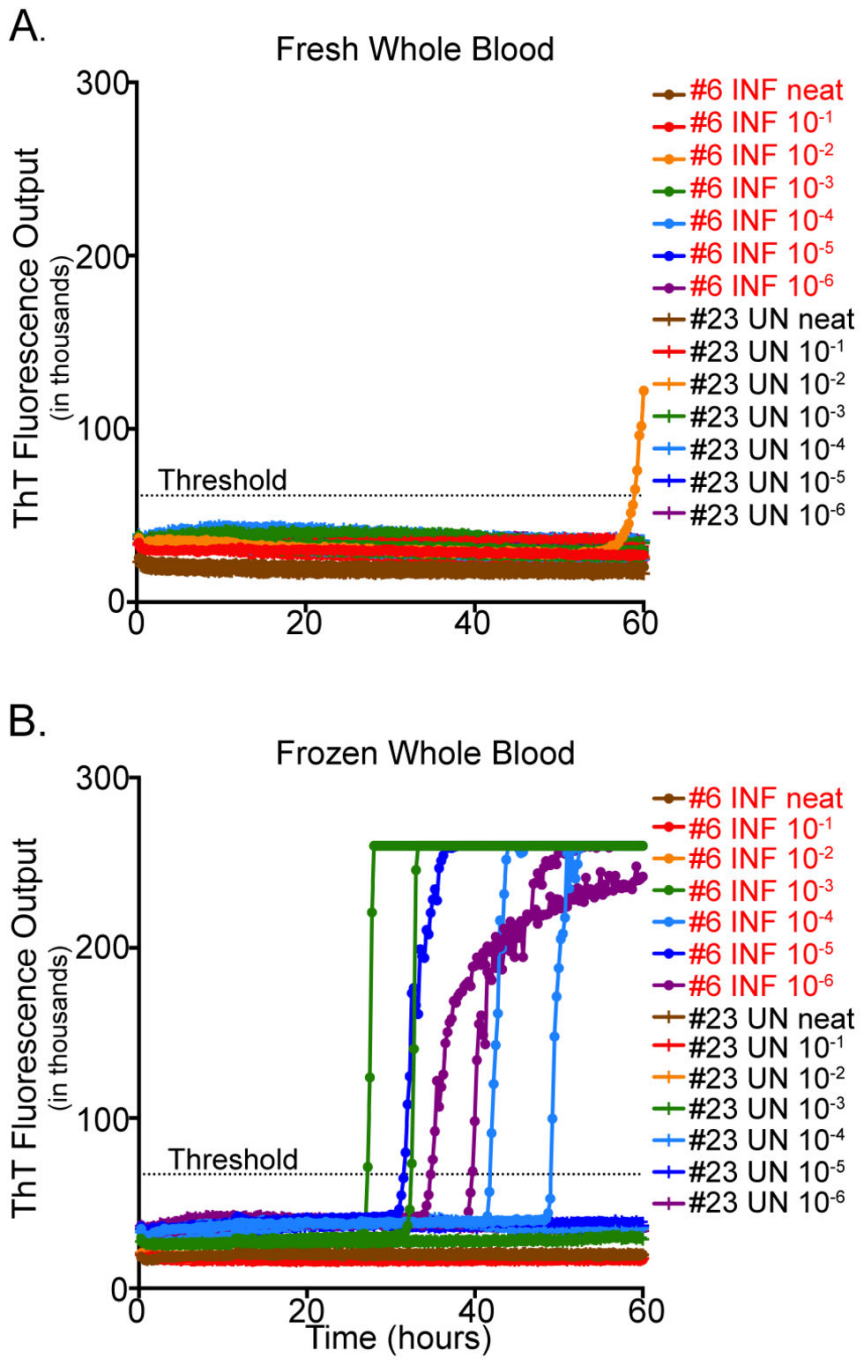
RT-QuIC analysis of fresh versus frozen whole blood. Blood was collected from a CWD-infected and CWD-naïve white-tailed deer and aliquots were analyzed immediately (fresh) or frozen (-80C). Serial blood sample dilutions (neat to 10^-6^) were assayed by RT-QuIC in duplicate for 60 hours and ThT fluorescence level above threshold determined positivity. Detection of PrP^C^-converting activity for each replicate is shown for blood analyzed fresh (A) and frozen (B).

In order to determine if historical blood samples were adequately preserved to initiate PrP^C^-converting activity in RT-QuIC, whole blood was collected from contemporary naïve and CWD-infected white-tailed deer and compared as fresh versus frozen samples. Samples were processed in various dilutions ranging from undiluted to 10^-6^ to determine the optimal dilution for PrP^D^ detection using frozen whole blood in the RT-QuIC assay. While PrP^C^-converting activity was detected in fresh whole blood, blood that had been processed through the freeze-thaw procedure yielded higher and more consistent detection of PrP^C^-converting activity (2/2 replicates in the 10^-3^, 10^-4^ and 10^-6^ dilutions; 1/2 replicates in the 10^-5^ dilution) (Figure 2). PrP^C^-converting activity was not observed in wells containing only substrate or naïve cervid blood. To determine if the results observed in the anticoagulant study were due solely to the use of fresh blood, the experiments were repeated on frozen blood collected in all three anticoagulants. Results revealed identical outcomes for both CPDA and EDTA blood while showing an increased sensitivity in heparin, as described above (data not shown). All subsequent RT-QuIC analyses included heparin-preserved whole blood that had undergone four freeze-thaw cycles.

### Effects of sodium phosphotungstic acid precipitation (NaPTA) on RT-QuIC PrP^D^ detection

**Figure 3:**
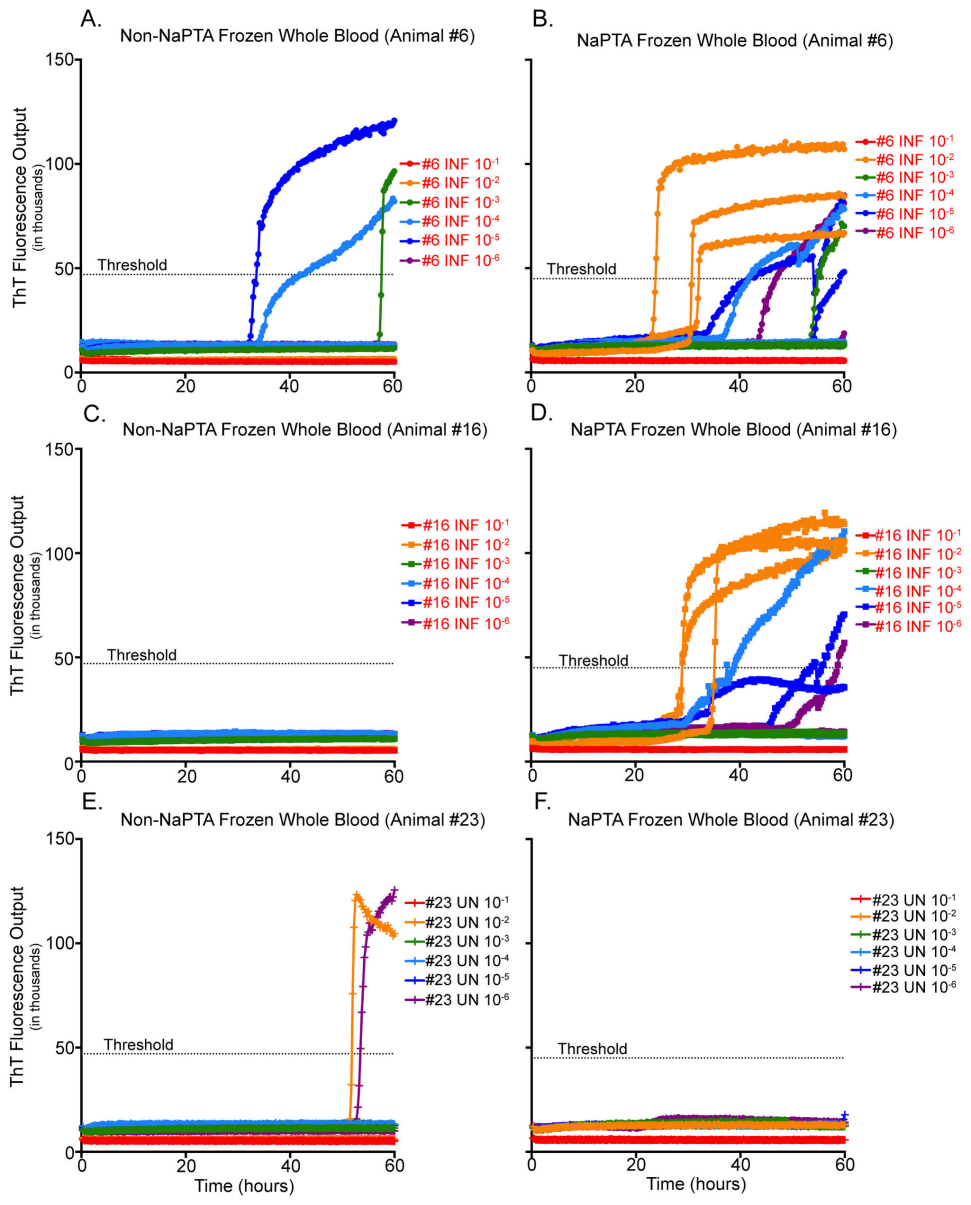
RT-QuIC analysis of samples before and after treatment with NAPTA. Samples were untreated or concentrated using NAPTA, serially diluted (neat to 10^-6^), and assayed by RT-QuIC in triplicate for 60 hours. ThT fluorescence level above threshold determined positivity, each replicate is present. (A and C) Limited detection is seen in untreated blood samples. (B and D) Improved detection of PrP^C^-converting activity is seen in blood samples precipitated with NAPTA from CWD-infected white-tailed deer. (E) Note increased false-positives in untreated samples from a CWD-naïve white-tailed deer. (F) No PrP^C^-converting activity was seen in samples precipitated with NAPTA from a CWD-naïve white-tailed deer.

While blood that had been freeze-thawed revealed more PrP^C^-converting activity than fresh blood, the results demonstrated an inconsistency in regards to the time required for a sample to become positive and dilutions that were positive. In addition to these inconsistencies, false-positive results were also witnessed. NaPTA precipitation was applied to heparin preserved whole blood that had undergone freeze-thaw cell lysis in an attempt to increase consistency of positive samples, as well as the sensitivity and specificity of the RT-QuIC assay. With the improved sensitivity and specificity provided by NaPTA pretreatment, we were able to demonstrate reliable RT-QuIC results at a 10^-2^ dilution of CWD-infected whole blood, while NaPTA treated whole blood from a naïve individual remained conversion free (Figure 3). Samples were serially diluted, with the dilutional series for each animal being run in triplicate for 60 hours. 

All of the remaining RT-QuIC analyses of TSE prion converting activity in historical and contemporary samples were conducted with heparin-preserved and freeze-thawed NaPTA-treated whole blood. 

### RT-QuIC comparison of CWD-positive brain versus NaPTA concentrated whole blood

**Figure 4:**
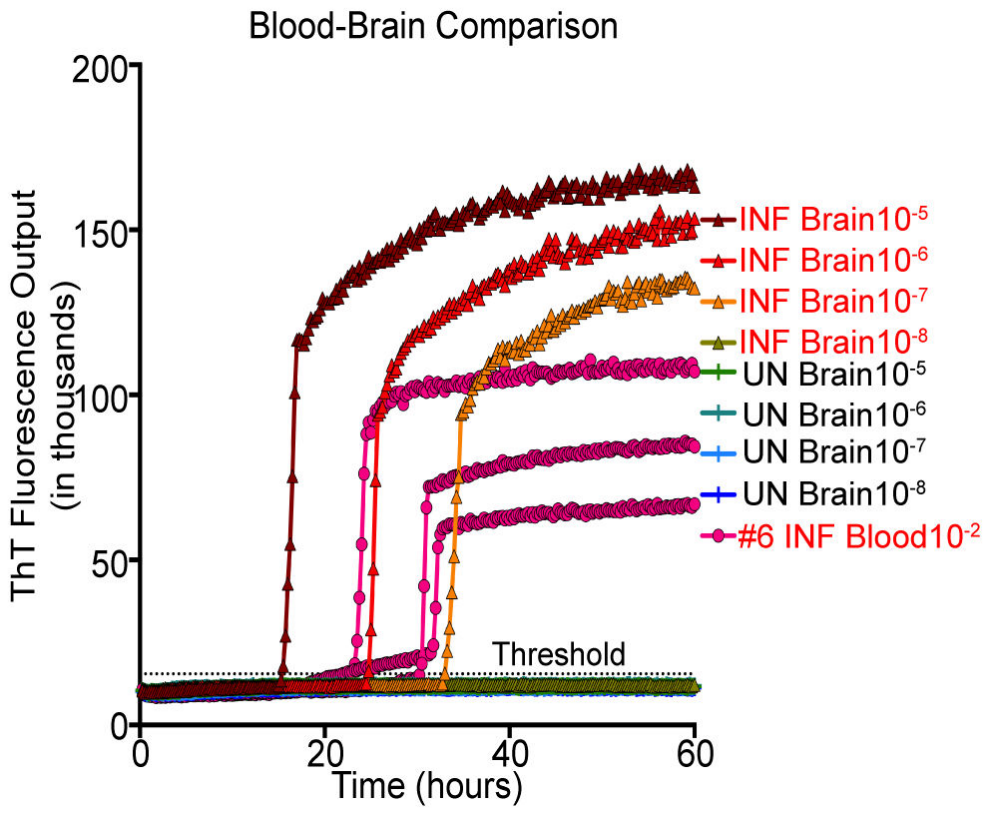
RT-QuIC comparison of brain and blood samples. Ten percent (10%) brain homogenates were serially diluted (10^-5^ to 10^-8^) and assayed by RT-QuIC for 60 hours. Blood samples were diluted to 10^-2^ and run in triplicate for 60 hours with ThT fluorescence level above threshold determining positivity. CWD-infected blood diluted 10^-2^ is seen to have similar levels of PrP^C^ converting activity as CWD-positive brain diluted 10^-6^ and 10^-7^. UN= Uninfected; INF= Infected.

To evaluate the levels of PrP^D^ present in NaPTA concentrated whole blood samples, PrP^C^-converting activity was compared to that detected in serial dilutions of CWD-positive white-tailed deer brain (Figure 4). NaPTA treated whole blood (500 µl starting volume of whole blood concentrated to 50 µl) diluted to 10^-2^ demonstrated PrP^D^ levels approximately equivalent to that measured in 10^-6^-10^-7^ dilution of CWD-positive brain. Equivalence was determined by comparison of the time to positivity for whole blood and brain samples.

### Detection of PrP^C^-converting activity in CWD-infected cervid whole blood

**Figure 5:**
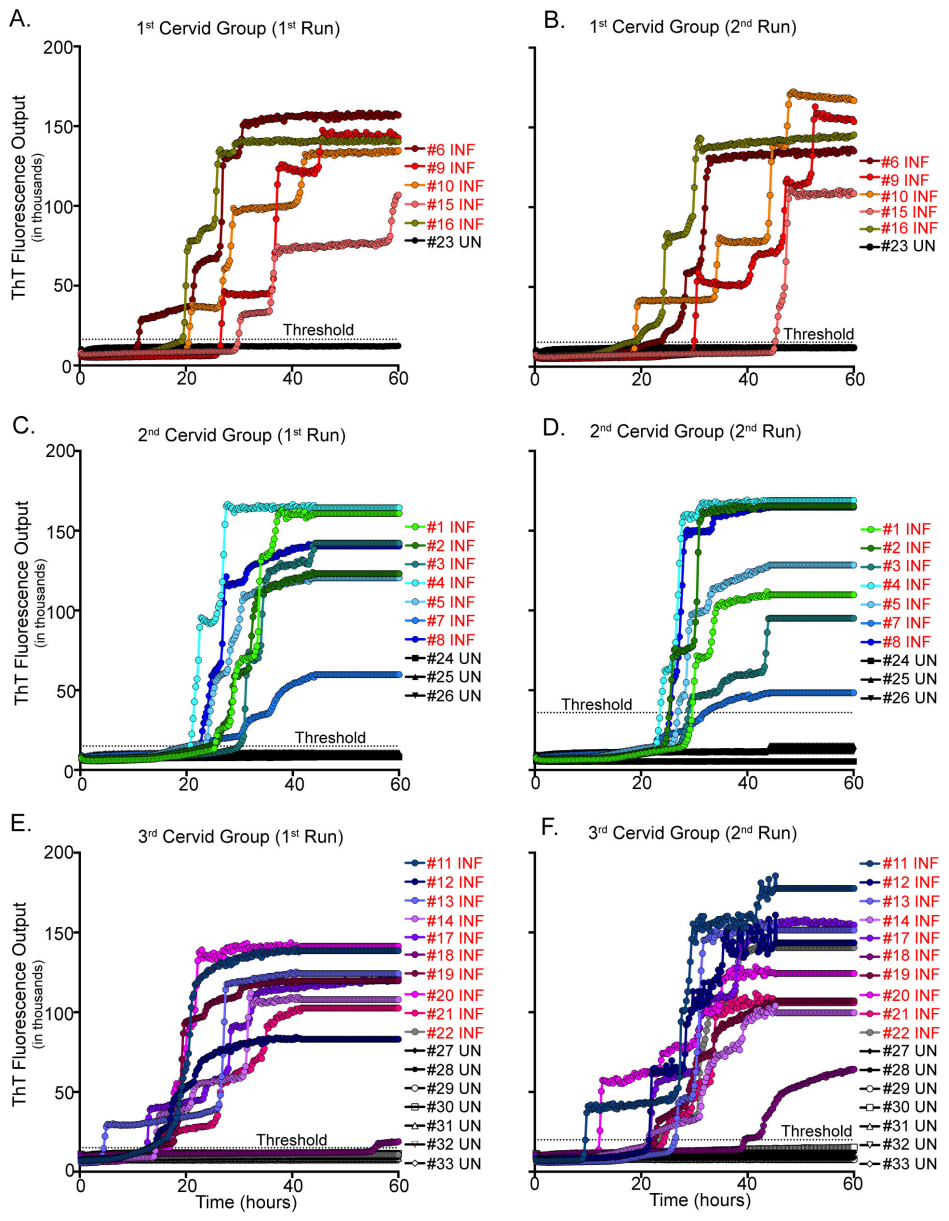
RT-QuIC analysis of cervid whole blood samples. Blood samples were diluted to 10^-2^ and 8 replicates were analyzed over 2 runs of 60 hours, and positivity was determined by ThT fluorescence level above threshold. PrP^C^-converting activity is demonstrated in 22 CWD-infected cervid blood samples, and is absent in all CWD-naïve samples (A-F). Each line is the average of four replicates for a specific animal. UN= Uninfected; INF= Infected.

Twenty-two of 22 clinical and preclinical CWD-infected cervids (16 white-tailed deer and 6 muntjac deer) and 0/11 naive cervids (5 white-tailed deer and 6 muntjac deer) exhibited RT-QuIC PrP^C^-converting activity in 7/8 or 8/8 replicates within 60 hours (Figure 5, Table 1). Samples were run two separate times to determine consistency of the RT-QuIC assay. Sample replicates were averaged on each plate and a positive threshold was set at five times the standard deviation of the negative control average.

**Table 1:** Cervid blood donor inoculation, clinical status, and assay results.

**Animal #**	**Inoculum**	**Route of Inoculation**	**Sample collection date**	**Disease Status**	**Western Blot Status (Obex)**	**IHC Status**	**Positive QuIC Replicates**
**1 (WTD**)	**2 ml 5% CWD+ brain homogenate**	**Aerosol**	**23 MPI**	**Clinical**	**+**	**+^B^**	**8/8**
**2 (WTD**)	**2 ml 5% CWD+ brain homogenate**	**Aerosol**	**22 MPI**	**Clinical**	**+**	**+^B^**	**8/8**
**3 (WTD**)	**2 ml 5% CWD+ brain homogenate**	**Aerosol**	**22 MPI**	**Clinical**	**+**	**+^B^**	**7/8**
**4 (WTD**)	**2 ml 5% CWD+ brain homogenate**	**Aerosol**	**23 MPI**	**Clinical**	**+**	**+^B^**	**8/8**
**5 (WTD**)	**2 ml 5% CWD+ brain homogenate**	**Aerosol**	**19 MPI Termination**	**Pre-clinical**	**+**	**+^B^**	**8/8**
**6 (WTD**)	**2 ml 5% CWD+ brain homogenate**	**Aerosol**	**16.5 MPI Termination**	**Clinical**	**+**	**+^B^**	**8/8**
**7 (WTD**)	**1.0 g 10% CWD+ brain homogenate**	**PO**	**24.5 MPI**	**Pre-clinical**	**NA**	**+^B^**	**7/8**
**8 (WTD**)	**1.0 g 10% CWD+ brain homogenate**	**PO**	**22 MPI Termination**	**Pre-clinical**	**+**	**+^B^**	**8/8**
**9 (WTD**)	**1.0 g 10% CWD+ brain homogenate**	**PO**	**22 MPI**	**Pre-clinical**	-	**-^B^**	**7/8**
**10 (WTD**)	**1.0 g 10% CWD+ brain homogenate**	**PO**	**22 MPI Termination**	**Clinical**	**+**	**+^B^**	**8/8**
**11 (WTD**)	**1.0 g 10% CWD+ brain homogenate**	**PO**	**16 MPI Termination**	**Pre-clinical**	**+**	**+^B^**	**8/8**
**12 (WTD**)	**1.0 g 10% CWD+ brain homogenate**	**PO**	**18 MPI Termination**	**Clinical**	**+**	**+^B^**	**7/8**
**13 (WTD**)	**1.0 g 10% CWD+ brain homogenate**	**PO**	**22 MPI Termination**	**Clinical**	**+**	**+^B^**	**8/8**
**14 (WTD**)	**1.0 g 10% CWD+ brain homogenate**	**PO**	**16 MPI Termination**	**Pre-clinical**	**+**	**+^B^**	**7/8**
**15 (WTD**)	**2.0 g 10% CWD+ brain homogenate**	**IC**	**12 MPI Termination**	**Clinical**	**+**	**+^B^,^O^**	**7/8**
**16 (WTD**)	**250 ml CWD+ whole blood**	**IV**	**12 MPI**	**Clinical**	**+**	**+^B^,^O^**	**8/8**
**17 (MJ**)	**1.0 g 10% CWD+ brain homogenate**	**PO/SQ**	**6.5 MPI Termination**	Pre-clinical*	-	**+^O^**	**8/8**
**18 (MJ**)	**1.0 g 10% CWD+ brain homogenate**	**PO/SQ**	**6.5 MPI Termination**	Pre-clinical*	-	**+^O^**	**6/8**
**19 (MJ**)	**1.0 g 10% CWD+ brain homogenate**	**PO/SQ**	**26 MPI Termination**	**Clinical**	**+**	**+^B^,^O^**	**8/8**
**20 (MJ**)	**1.0 g 10% CWD+ brain homogenate**	**PO/SQ**	**23 MPI Termination**	**Clinical**	**+**	**+^B^,^O^**	**8/8**
**21 (MJ**)	**1.0 g 10% CWD+ brain homogenate**	**PO/SQ**	**22 MPI Termination**	**Clinical**	**+**	**+^B^,^O^**	**8/8**
**22 (MJ**)	**1.0 g 10% CWD+ brain homogenate**	**PO/SQ**	**24 MPI Termination**	**Clinical**	**+**	**+^B^,^O^**	**4/8**
**23 (WTD**)	**2 ml sham homogenate**	**Aerosol**	**19 MPI**	**NA**	-	**-^B^**	**0/8**
**24 (WTD**)	**2 ml sham homogenate**	**Aerosol**	**23 MPI**	**NA**	-	**-^B^**	**0/8**
**25 (WTD**)	**2 ml sham homogenate**	**Aerosol**	**22 MPI**	**NA**	-	**-^B^**	**0/8**
**26 (WTD**)	**Uninfected urine/feces**	**PO**	**20 MPI**	**NA**	-	**-^B,O^**	**0/8**
**27 (WTD**)	**Uninfected urine/feces**	**PO**	**20 MPI**	**NA**	-	**-^B,O^**	**0/8**
**28 (MJ**)	**1.0 g sham homogenate**	**PO/SQ**	**13 MPI**	**NA**	-	**-^O^**	**0/8**
**29 (MJ**)	**1.0 g sham homogenate**	**PO/SQ**	**23 MPI**	**NA**	-	**-^O^**	**0/8**
**30 (MJ**)	**Uninoculated**	**NA**	**NA**	**NA**	-	**-^O^**	**0/8**
**31 (MJ**)	**Uninoculated**	**NA**	**NA**	**NA**	-	**-^O^**	**0/8**
**32 (MJ**)	**Uninoculated**	**NA**	**NA**	**NA**	-	**-^O^**	**0/8**
**33 (MJ**)	**Uninoculated**	**NA**	**NA**	**NA**	-	**-^O^**	**0/8**

**WTD = White-tailed deer**

**MJ = Muntjac deer**

**NA = Not available**

**- = PrP^D^ was not detected in the sample**

**+ = PrP^D^ was detected in the sample**

**MPI = Months post inoculation**

**^B^ = Biopsy of tonsil and recto-anal mucosa associated lymphoid tissue**

**^O^ = Obex**

* Less than halfway to clinical disease

### Detection of PrP^C^-converting activity in TME-infected hamster whole blood

**Figure 6:**
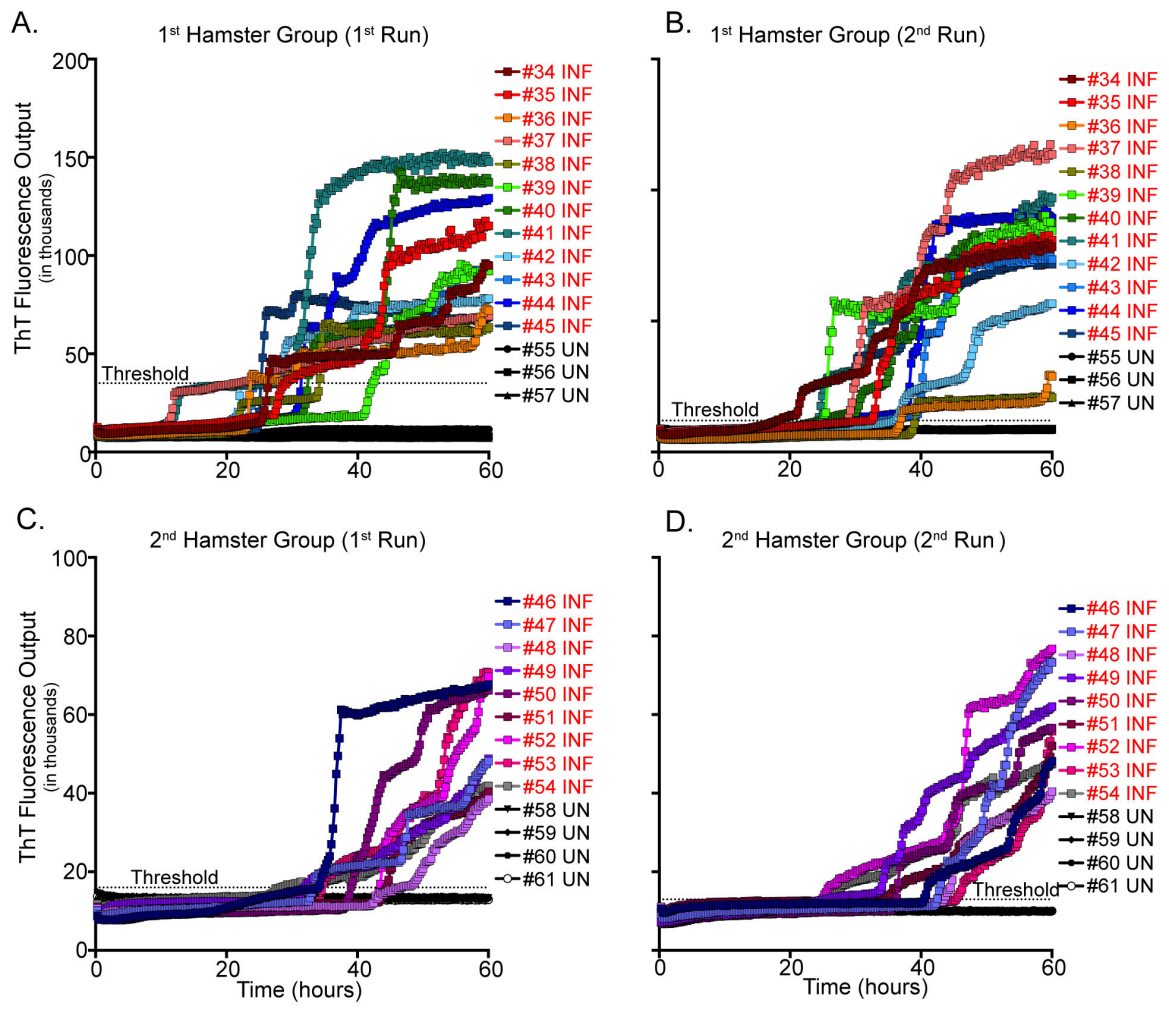
RT-QuIC analysis of hamster whole blood samples. Blood samples were diluted to 10^-2^ and 8 replicates were analyzed over 2 experiments of 60 hours, and positivity was determined by ThT fluorescence level above threshold. PrP^C^-converting activity is demonstrated in 21 TME-infected blood samples, and is absent in all TME-naïve samples (A-D). Each line is the average of four replicates for a specific animal. UN= Uninfected; INF= Infected.

The hyper strain of transmissible mink encephalopathy (HY TME) was chosen for the RT-QuIC assay to determine the assays ability for PrP^D^ detection in various species and strains of TSEs. All HY TME-infected hamsters (n=21), ranging from 8 to 20 weeks post infection, exhibited RT-QuIC PrP^C^-converting activity in 5/8 - 8/8 replicates within 60 hours, while all (n=7) of the age matched controls failed to seed RT-QuIC (Figure 6, Table 2). As above, each sample was run two separate times to determine consistency of the RT-QuIC assay. Sample replicates were averaged on each plate and a positive threshold was set at five times the standard deviation of the negative control average. 

**Table 2:** Hamster blood donor inoculation, clinical status, and assay results.

**Animal #**	**Inoculum**	**Route of inoculation**	**Disease Status**	**Sample collection date**	**IHC Status**	**Positive QuIC Replicates**
**34**	**10 µl 10% HY TME brain homogenate**	**Extranasal**	Pre-clinical*	**8 WPI**	**ND**	**8/8**
**35**	**10 µl 10% HY TME brain homogenate**	**Extranasal**	Pre-clinical*	**8 WPI**	**ND**	**8/8**
**36**	**10 µl 10% HY TME brain homogenate**	**Extranasal**	Pre-clinical*	**8 WPI**	**ND**	**5/8**
**37**	**10 µl 10% HY TME brain homogenate**	**Extranasal**	Pre-clinical*	**10 WPI**	**ND**	**8/8**
**38**	**10 µl 10% HY TME brain homogenate**	**Extranasal**	Pre-clinical*	**10 WPI**	**ND**	**5/8**
**39**	**10 µl 10% HY TME brain homogenate**	**Extranasal**	Pre-clinical*	**10 WPI**	**ND**	**8/8**
**40**	**10 µl 10% HY TME brain homogenate**	**Extranasal**	**Pre-clinical**	**12 WPI**	-	**8/8**
**41**	**10 µl 10% HY TME brain homogenate**	**Extranasal**	**Pre-clinical**	**12 WPI**	-	**8/8**
**42**	**10 µl 10% HY TME brain homogenate**	**Extranasal**	**Pre-clinical**	**12 WPI**	-	**8/8**
**43**	**10 µl 10% HY TME brain homogenate**	**Extranasal**	**Pre-clinical**	**14 WPI**	-	**8/8**
**44**	**10 µl 10% HY TME brain homogenate**	**Extranasal**	**Pre-clinical**	**14 WPI**	**+**	**8/8**
**45**	**10 µl 10% HY TME brain homogenate**	**Extranasal**	**Pre-clinical**	**14 WPI**	**+**	**8/8**
**46**	**10 µl 10% HY TME brain homogenate**	**Extranasal**	**Pre-clinical**	**16 WPI**	**+**	**8/8**
**47**	**10 µl 10% HY TME brain homogenate**	**Extranasal**	**Pre-clinical**	**16 WPI**	**+**	**7/8**
**48**	**10 µl 10% HY TME brain homogenate**	**Extranasal**	**Pre-clinical**	**16 WPI**	**+**	**8/8**
**49**	**10 µl 10% HY TME brain homogenate**	**Extranasal**	**Pre-clinical**	**18 WPI**	**ND**	**8/8**
**50**	**10 µl 10% HY TME brain homogenate**	**Extranasal**	**Pre-clinical**	**18 WPI**	**ND**	**7/8**
**51**	**10 µl 10% HY TME brain homogenate**	**Extranasal**	**Pre-clinical**	**18 WPI**	**ND**	**8/8**
**52**	**10 µl 10% HY TME brain homogenate**	**Extranasal**	**Clinical**	**20 WPI**	**ND**	**8/8**
**53**	**10 µl 10% HY TME brain homogenate**	**Extranasal**	**Clinical**	**20 WPI**	**ND**	**7/8**
**54**	**10 µl 10% HY TME brain homogenate**	**Extranasal**	**Clinical**	**20 WPI**	**ND**	**8/8**
**55**	**10 µl 10% sham homogenate**	**Extranasal**	**NA**	**8 WPI**	-	**0/8**
**56**	**10 µl 10% sham homogenate**	**Extranasal**	**NA**	**10 WPI**	-	**0/8**
**57**	**10 µl 10% sham homogenate**	**Extranasal**	**NA**	**12 WPI**	-	**0/8**
**58**	**10 µl 10% sham homogenate**	**Extranasal**	**NA**	**14 WPI**	-	**0/8**
**59**	**10 µl 10% sham homogenate**	**Extranasal**	**NA**	**16 WPI**	-	**0/8**
**60**	**10 µl 10% sham homogenate**	**Extranasal**	**NA**	**18 WPI**	-	**0/8**
**61**	**10 µl 10% sham homogenate**	**Extranasal**	**NA**	**20 WPI**	-	**0/8**

**WPI = weeks post inoculation**

**NA = Not available**

**ND = Not done**

**- = PrP^D^ was not detected**

**+ = PrP^D^ was detected in the sample**

* = Less than/equal to the halfway point to clinical disease

### Immunohistochemistry confirmation of RT-QuIC results

**Figure 7:**
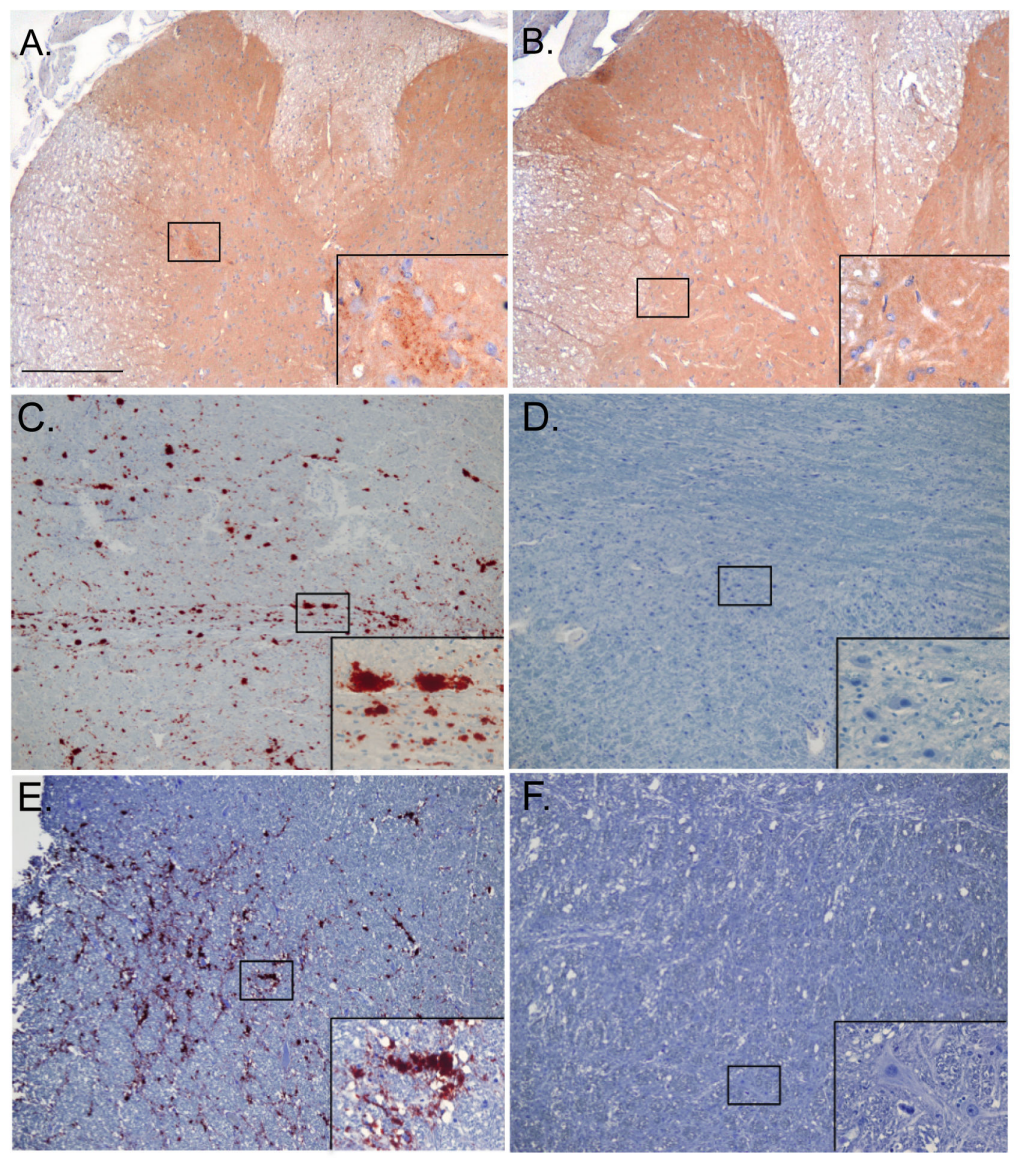
PrP^D^ detection in hamster, white-tailed deer and muntjac by IHC. PrP^D^ immunoreactivity in a spinal cord tissue section from a hamster 16 weeks after extranasal inoculation with HY-TME (A) detected with antibody 3F4 and ABC solution. PrP^D^ immunoreactivity in the brainstem of CWD-infected white-tailed deer (C) and muntjac (E) detected with antibody BAR224 and AEC substrate. No immunoreactivity was seen in the corresponding tissues of mock-inoculated controls (B, D and F). The boxed areas are enlarged 10x in the insets. Scale bar = 200µm.

Immunohistochemistry was applied as a confirmation for the presence of PrP^D^ deposition in the brains of animals where PrP^C^-converting activity was detected in blood. IHC was performed on both cervid and hamster TME-inoculated and mock-inoculated brains for detection of the disease associated isoform of the prion protein, PrP^D^. PrP^D^ deposition was observed in TSE-infected animals, but not in mock-inoculated animals (Figure 7; Tables 1, 2).

### Mouse and hamster bioassay sensitivity vs. RT-QuIC sensitivity

To determine the brain equivalent sensitivity of RT-QuIC for TME and CWD samples, RT-QuIC analysis of serial dilutions of TSE-positive brain homogenates were compared to lethal dose bioassay titrations in HY TME-infected hamsters and CWD-infected mice.

**Figure 8:**
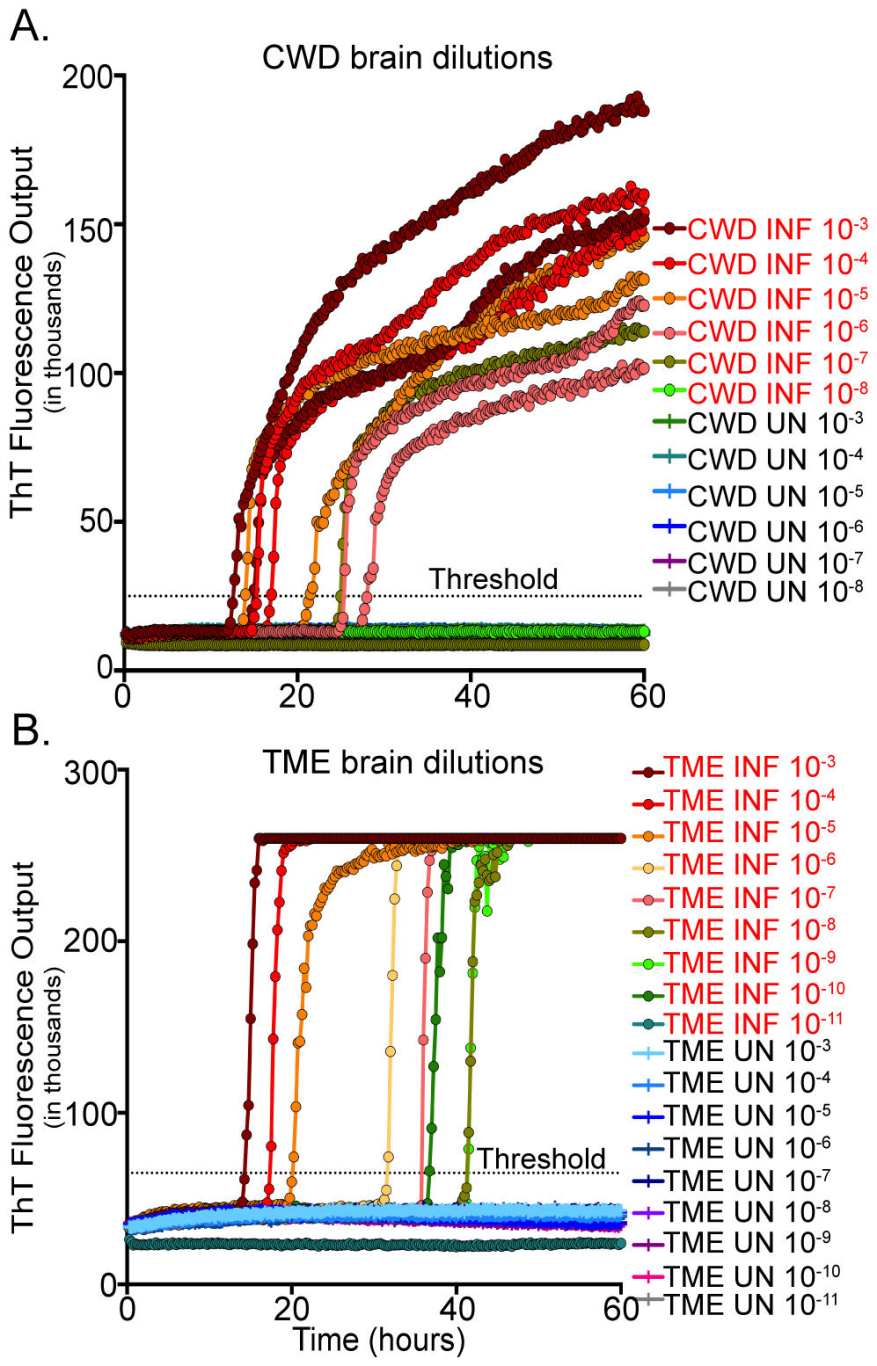
RT-QuIC analysis of serially diluted cervid and hamster brain samples. Brain samples were serially diluted 10^-3^ to 10^-6^ or 10^-3^ to 10^-10^ for cervids (A) and hamsters (B), respectively, and analyzed in RT-QuIC for 60 hours. A ThT fluorescence level above threshold determined positivity. Both cervid and hamster brains from positively inoculated animals demonstrated positivity in all dilutions, while all brain dilutions from naïve animals remained negative.

Using bioassay in cervidized transgenic mice and the Reed-Muench method, the LD_50_ titer for 1 ml of 10% CWD-positive brain homogenate was determined to be 10^4.664^, or 4.62x10^4^ units/ml (calculated from values in Table 3). End point dilution analysis revealed a failure to cause disease in dilutions greater than 10^-5^. Serial dilutions of 10% homogenate CWD-positive brain homogenates in RT-QuIC demonstrated consistent positivity to a dilution of 10^-6^, with 50% converting activity detected in the 10^-7^ dilution (Figure 8A). SD_50_ titer for the RT-QuIC assay was calculated for 1 ml of CWD-positive brain and was determined to be 10^9.544^, or 3.5x10^9^ units/ml. These results indicate that the sensitivity of RT-QuIC for CWD detection is greater than animal bioassay.

**Table 3:** Bioassay of CWD-positive cervid brain in TgCerPrP mice.

**Dose (% brain homogenate)**	**# Clinical/total n**	**Days post inoculation (DPI) to clinical disease**
**10**	**7/9^A^**	**137 ± 63 DPI**
**1**	**9/9**	**200 ± 29 DPI**
**0.1**	**8/9^B^**	**220 ± 70 DPI**
**0.01**	**8/9^B^**	**250 ± 68 DPI**
**0.001**	**7/9**	**397 ± 152 DPI**
**0.0001**	**1/9**	**335 DPI**
**0.00001**	**0/9**	**NA**

**DPI = Days post inoculation**

**^A^ = 2/9 mice died for reasons unrelated to CWD infection**

**^B^ = 1/9 mice died for reasons unrelated to CWD infection**

**NA = Not applicable**

The LD_50_ for hamsters intracranialy inoculated with HY TME was determined to be 10^9.3^, as demonstrated previously by Kincaid, et al. [32]. Endpoint dilution analysis resulted in failure to cause disease in dilutions greater than 10^-9^. RT-QuIC analysis of the same HY TME brain homogenates revealed PrP^C^-converting activity to 10^-10^ (Figure 8B). SD_50_ titer for the RT-QuIC assay was calculated for 1 ml of HY TME-positive brain and was determined to be 10^13.033^, or 1.08x10^13^ units/ml. This indicates that the sensitivity of RT-QuIC for HY TME detection is greater than animal bioassay. 

## Discussion

### RT-QuIC analysis of whole blood collected in various anticoagulants

Precedence for hematogenous spread of prions via transfusion has been well established with various TSEs, including: scrapie [11], CWD [13,33], BSE in sheep [10] and vCJD [14,16-19]. To date, few *in vitro* assays are capable of detecting prions present in the blood of infected individuals, and those that do can suffer from decreased sensitivity, possibly due to the presence of assay inhibitors [26,34-36].

To assess whether anticoagulants affect PrP^D^ detection, we analyzed whole blood collected in CPDA, EDTA, and heparin. It has been demonstrated in previous work [37] that anticoagulant storage can affect the presentation of cellular PrP. Here, we have shown that whole blood collected in heparin, but not in CPDA or EDTA, elicited efficient *in vitro* RT-QuIC prion conversion. In addition to the conversion observed in heparin-preserved whole blood, it should be noted that only more dilute samples (10^-5^ dilution in particular) demonstrated PrP^C^-converting activity. We suspect that this is due to the presence of inhibitory products in whole blood and that further diluting samples aids in the removal of these inhibitory products.

It has been shown that polyanions enhance the amplification of prions in *in vitro* conversion assays, suggesting that they may contribute to conversion efficiency [38,39]. Heparin, a polyanion, has previously been shown to enhance *in vitro* detection of PrP^D^ [40] and is thought to serve as a potential cofactor in prion propagation *in vivo* by acting as a scaffolding molecule or catalyst due to its highly negatively charged-glycosaminoglycan nature [41]. EDTA and CPDA owe their anticoagulant property to their ability to chelate calcium in blood. Chelating agents are widely used for scavenging metal ions [42] which may contribute to the absence of PrP^C^-conversion observed in blood collected in the two anticoagulants. However, it should be noted that detection of prions in CPDA blood has been observed following immunoaffinity capture and substrate replacement [29]. Further research is needed to determine the role anticoagulants play in inhibiting/facilitating RT-QuIC. We have demonstrated that preserving whole blood samples in heparin may facilitate *in vitro* prion detection.

### RT-QuIC analysis of fresh versus frozen whole blood

To assess the feasibility of using historical frozen samples for future analysis of blood-borne prions, we evaluated the effects of freezing blood prior to RT-QuIC. We have demonstrated that the freeze-thaw cycle enhances RT-QuIC blood-borne prion detection sensitivity, facilitating *in vitro* prion detection at earlier time points with a more robust amplification than samples that did not undergo the freeze-thaw process. There is compelling evidence for the accumulation of aggregated misfolded prion isoforms in the cytoplasm of infected cells [43,44] and it is hypothesized that these aggregates are released from the cell as lysis occurs. Thermal shock on whole blood samples damages the cell membrane and initiates hemolysis [45,46], which is thought to release intracellular components. Cell lysis of blood collected from TSE-infected animals, associated with repeated freeze-thaw cycles, may liberate sufficient prions to enhance *in vitro* nucleation and thus the detection of PrP^C^-converting activity. 

### Effects of sodium phosphotungstic acid precipitation on RT-QuIC PrP^D^ detection

It has been suggested that there are components present in bodily fluids that interfere with or inhibit prion conversion and thus *in vitro* detection of the aberrant form of the prion protein [47,48]. Various groups have attempted to solve this problem using different concentration methods. Using immunoprecipitation coupled with RT-QuIC, Orrú et al. [29] were able to establish *in vitro* detection of PrP^C^-converting activity in plasma and serum samples from scrapie-infected hamsters. Morales et al. [49] demonstrated that the use of varying concentrations of sarkosyl could concentrate PrP^D^ present in tissue and fluid samples. Wadsworth and colleagues [50,51] have shown that sarkosyl, coupled with the use of sodium phosphotungstic acid, enhances the isolation of both PrP^C^ and PrP^D^ from bodily fluids. Some groups have reported an inhibitory effect on amyloid formation when using NaPTA precipitation [52]; however, this was not our experience (Figure 3). 

Using NaPTA precipitation we were able to concentrate hematogenous prions to a more detectable level and/or remove assay inhibitors, augmenting our ability to directly detect prions in whole blood. Samples not receiving NaPTA treatment took longer to convert PrP^C^, and only in more dilute samples (Figure 3A). Samples that received treatment with NaPTA precipitation revealed PrP^C^-converting activity earlier, and exhibited positivity in more concentrated samples (Figure 3B). We conclude that NaPTA precipitation may remove potential assay inhibitors that are present in blood, allowing detection of converting activity at more concentrated dilutions thus decreasing false negatives (Figure 3C, D). In addition to these observations, we attempted sonication of the NaPTA product prior to serial dilution to determine if this aided in the observation of a dose-response. While this method slightly increased the number of later dilutions expressing PrP^C^-converting activity and aided in the consistency of when they crossed the positivity threshold (data not shown), the effect witnessed was not great enough to alter our decision to use the 10^-2^ dilution for all subsequent experiments.

With the application of an anticoagulant that facilitates prion conversion *in vitro*, the freeze-thaw cell lysis and NaPTA precipitation, we have optimized the RT-QuIC assay for efficient detection of PrP^D^ in whole blood samples, thus we are calling our new protocol whole blood optimized (WBO) RT-QuIC. NaPTA precipitation increased consistency, the number of positive replicates and decreased the assay time required to initiate PrP^C^ conversion/detection in whole blood harvested from TSE-infected animals while limiting false positive PrP^C^-converting activity in samples from uninfected animals (Figure 3). 

### RT-QuIC comparison of CWD-positive brain and NaPTA concentrated whole blood

Many groups have developed quantitative *in vitro* methods to analyze the levels of PrP^D^ present in various tissues and bodily fluid samples. Murayama et al. [53] used PMCA to establish a direct comparison of PrP^D^ levels in buffy coat and plasma to PrP^D^ levels seen in serial dilutions of TSE-infected brain by analyzing which round of PMCA samples began demonstrating positivity. Other laboratories [26,48,54] have reported quantitative and semi-quantitative methods of PMCA to determine the levels of PrP^D^ in blood and urine by comparing to the amount of amplifiable PrP^D^ present in TSE-infected brain. Castilla et al. [26] were able to demonstrate that PMCA amplifiable prions in buffy coat collected from 1 ml of scrapie-adapted hamster blood contained roughly 0.1-1 pg of PrP^D^ molecules. Our RT-QuIC results indicate that 2 µl of a 10^-2^ dilution (0.5 ml of whole blood NaPTA concentrated 10-fold, further diluted to 10^-2^) contained PrP^D^ levels equivalent to those seen in 0.02 ng - 0.2 ng of CWD-positive brain (Figure 4). 

### Detection of PrP^C^-converting activity in CWD-infected cervid whole blood

Wilham et al. [30] demonstrated that the RT-QuIC assay has the ability to detect prions in tissue samples with similar sensitivity as bioassay (~ 1 lethal dose), rendering it appropriate for the detection of PrP^D^ in bodily fluids such as blood and saliva. RT-QuIC assay efficacy for CWD-infected whole blood was evaluated following pretreatment to augment the release of prions from carrier cells and minimize inhibitory factors (freeze-thaw/NaPTA). We have demonstrated that our optimized RT-QuIC assay is sufficiently sensitive to detect PrP^C^-converting activity in whole blood harvested from preclinical and clinical IHC/Western blot-confirmed CWD-infected animals. Furthermore, our optimized RT-QuIC assay has demonstrated the ability to detect PrP^C^-converting activity in CWD-inoculated animals prior to the mid point between inoculation and clinical disease.

Using PMCA for the detection of PrP^D^ in the blood of scrapie-infected hamsters, Saa et al. [25] reported sensitivity levels of 80% for clinical animals, and up to 60% for preclinical animals. Orrú et al. demonstrated even greater sensitivity for PrP^D^ in blood plasma of scrapie-infected hamsters using immunoprecipitation coupled with RT-QuIC [29]. Utilizing our optimized RT-QuIC assay for cervid whole blood, we have shown that our assay exhibited sensitivity levels of 93.8% for clinical animals and 92.2% for preclinical animals while maintaining 100% specificity. These results reveal the potential of RT-QuIC as a reliable *in vitro* assay for blood-borne prion detection.

### Detection of PrP^C^-converting activity in TME-infected hamster whole blood

Utilization of hamster models for the propagation and detection of hematogenous PrP^D^ have been used extensively [25,26,29,43,53,55], primarily with scrapie infections. Previous to this study, RT-QuIC had not been used to probe for PrP^C^-converting activity in whole blood of TME-infected hamsters. To ensure that the detection of RT-QuIC blood-borne PrP^D^ detection was not exclusive to CWD, we analyzed whole blood harvested from IHC-confirmed TME-infected and mock-infected hamsters. We have demonstrated PrP^C^-converting activity in preclinical TME-infected hamsters with 94.4% sensitivity and 100% specificity. We have also shown that the WBO RT-QuIC assay possesses the ability to detect PrP^D^ in the blood of TME-infected hamsters prior to the midpoint between inoculation and clinical disease.

These observations reveal that RT-QuIC is consistently more sensitive in detection of hematogenous PrP^D^ in preclinical animals than previously reported for PMCA [25]. Thus, the WBO RT-QuIC assay may be applicable for the detection of prionemia in multiple species (animals/humans).

### Implications for *in vitro* detection of blood-borne prion disease

The development of a reliable *in vitro* blood-borne TSE-detection assay would have significant advantages for both human and animal populations and may provide a stepping-stone for the development of diagnostic assays for other protein misfolding diseases. To date, various *in vitro* assays have been developed with the goal of detecting prions present in blood [56]. Of particular note are sPMCA [25,26], a ligand based assay developed to detect hematogenous prions [34], and immunoprecipitation enhanced RT-QuIC [29]. However, demonstrating satisfactory sensitivity and specificity with these assays has been a challenge.

We have demonstrated *in vitro* detection of prionemia in CWD and TME-infected hosts during both pre-clinical and clinical phases of disease, establishing the merits of RT-QuIC as an effective antemortem diagnostic tool. Early detection and screening applications will provide a means to detect asymptomatic carriers of TSE disease in the human donor blood and tissue-pools, thus indicating which samples should be eliminated. The ability to detect infected blood will aid in establishing monitoring parameters for TSE intervention/therapeutic strategies and provide domestic and wildlife herd management professionals with a live test for TSE surveillance. 

## Materials and Methods

### Ethics Statement

All animals were handled in strict accordance with guidelines for animal care and use provided by the United States Department of Agriculture (USDA), National Institutes of Health (NIH) and the Association for Assessment and Accreditation of Laboratory Animal Care International (AAALAC), and all animal work was approved by Colorado State University Institutional Animal Care and Use Committee (IACUC) Institutional Animal Care and Use Committee (IACUC) (approval numbers 02-151A, 08-175A and 11-2615A). All procedures involving hamsters were preapproved by the Creighton University Institutional Animal Care and Use Committee and were in compliance with the *Guide for the Care and Use of Laboratory Animals*.

### Cervid inoculations

Cervid whole blood was procured from historical and contemporary white-tailed and muntjac deer studies conducted at CSU (Table 1). Prior to inoculation, cervids were anesthetized with a mixture of ketamine and medetomidine. In brief, naïve 1-2 year old white-tailed deer (*Odocoileus virginianus*) were inoculated with CWD-positive material as follows: 1) 1.0 g of brain in a 10% brain homogenate (10 ml) administered intracranialy [13]; 2) 250 ml fresh/frozen whole blood administered intravenously/intraperitonealy, respectively [13]; 3) 1.0 g of brain in a 10% brain homogenate administered orally; or 4) 2 ml of a 5% (wt/vol) brain homogenate aerosol-administered [57]. Negative control white-tailed deer were exposed to sham inoculum as described above. Naïve 1-2 year old muntjac deer (*Muntiacus reevesi*) were inoculated with 1.0 g total brain in a 10% brain homogenate administered orally/subcutaneously [58]. Negative control muntjac deer received sham inoculum as described above.

### Hamster inoculation

Male 10-11 week old Syrian hamsters (Harlan Sprague Dawley, Indianapolis, IN) were used in these studies. Extranasal (e.n.) inoculations using a 10% w/v brain homogenate containing 10^6.8^ intracerebral 50% lethal doses per ml of the HY TME agent or a sham homogenate were performed as previously described [59]. Hamsters receiving e.n. inoculations were briefly anesthetized with isoflurane (Webster Veterinary), placed in a supine position and 5 µl of brain homogenate was placed just inferior to each nostril (10 µl total volume). Brain homogenate was immediately inhaled into the nasal cavity, as hamsters are obligate nose breathers.

### Blood and tissue collection from cervids

Whole blood (10 ml/cervid/anticoagulant) was collected from n=22 CWD-inoculated cervids following anesthetization with ketamine and medetomidine — six in various stages of disease presentation and 16 at termination— and from 11 negative control sham-inoculated cervids (Table 1). All blood samples were preserved in one of three anticoagulants: 1) 14% anticoagulant citrate phosphate dextrose adenine (CPDA), 2) 15% ethylenediaminetetraacetic acid (EDTA), or 3) 200 units/ml heparin, before being placed in 1 ml aliquots and frozen at -80°C. At termination cervids were euthanized with beuthanasia-D solution. Brain (medulla oblongata) collected from each terminal white-tailed deer and muntjac deer was frozen at -80°C or fixed in 10% neutral buffered formalin or paraformaldehyde-lysine-periodate (PLP) and stored in 60% ethanol prior to processing.

### Blood and tissue collection from hamsters

At selected time points post-infection, three infected and one mock-infected hamster were anesthetized with isoflurane and blood was collected via cardiac puncture into heparin blood tubes for preservation at -80°C (Table 2). The animals were then transcardially perfused with 50 ml of 0.01 M Dulbecco’s phosphate buffered saline followed by 75 ml of McLean’s PLP fixative. Brain and brainstem were immediately removed and placed in PLP for 5-7 hours at room temperature prior to paraffin processing and embedding.

### Brain tissue homogenization

Ten percent (10%) brain tissue homogenates were prepared from the obex region of the medulla oblongata by homogenizing 0.05 g brain tissue in 0.5 ml homogenate buffer (1X PBS + 0.1% Triton-X 100 [Sigma-Aldrich]). Samples were homogenized using 0.5 mm diameter zirconium oxide beads and a Bullet Blender (Next Advance) for 5 minutes at a speed setting of 10. Homogenates were stored at -80°C in 20 µl aliquots.

### Whole blood freeze-thaw and homogenization process

One milliliter (1 ml) aliquots of whole blood were frozen at -80°C for 30 minutes and subsequently thawed at 22°C for 60 minutes. This process was repeated four times. Samples were then homogenized using 0.5 mm diameter zirconium oxide beads and a Bullet Blender (Next Advance) for 5 minutes at top speed. 

### Sodium phosphotungstic acid (NaPTA) precipitation

Sodium phosphotungstic acid (NaPTA) precipitation of prions, as first described by Wadsworth et al. [50], was used to concentrate proteins (including PrP) present in whole blood samples. Frozen whole blood homogenates were thawed and centrifuged at 2000 rpm for one minute to remove cellular debris. Five hundred microliters (500 µl) of supernatant were mixed with an equal volume of 4% sarkosyl in 1X phosphate buffered saline (PBS) and incubated for 30 minutes at 37°C with constant agitation. Samples were then adjusted to contain a final concentration of 50 U/ml of benzonase (Sigma-Aldrich) and incubated at 37°C for another 30 minutes with constant agitation. A solution of 4% (w/v) phosphotungstic acid (Sigma-Aldrich) and 170 mM magnesium chloride, adjusted to pH 7.4 with NaOH, was added to the sample for a final concentration of 0.3% (w/v) NaPTA and agitated at 37°C for 30 minutes. Samples were then centrifuged for 30 minutes at 14,000 rpm and the pellet was resuspended in 50 µl 0.1% (v/v) sarkosyl.

### Recombinant protein preparation

Recombinant protein was expressed and purified as previously described [60,61]. Truncated recombinant Syrian hamster PrP (SHrPrP 90-231; received from the Caughey laboratory) expressed by Rosetta strain *Escherichia coli* was inoculated into 1 liter of LB containing Auto Induction^TM^ supplements (EMD Biosciences). Cultures were allowed to grow overnight until harvest when an OD (600nm) of ~3 was reached. Cells were lysed using Bug Buster^TM^ and Lysonase^TM^ (EMD Biosciences). Inclusion bodies (IB) were isolated by centrifugation at 15,000xg and were solubilized in 8 M guanidine hydrochloride in Tris-phosphate buffer (100 mM NaPO_4_ and 10 mM Tris pH 8.0). The protein solution obtained was bound to Super Flow Ni-NTA resin (Qiagen) pre-equilibrated with denaturing buffer (6.0 M GuHCl Tris-phosphate) at room temperature with agitation for 45 minutes and added to a XK FPLC column (GE). SHrPrP was refolded on the column with refolding Tris-phosphate buffer at 0.75 ml/min for 340 ml, then eluted with 0.5 M imidazole Tris-phosphate pH 5.5 at 2.0 ml/min for a total of 100 ml. Eluted fractions were collected and dialyzed in two changes of 4.0 liters dialysis buffer (20 mM NaPO_4_ pH 5.5). Following dialysis, purified protein was adjusted to 0.6 mg/ml, flash frozen in 1 ml aliquots, and stored at -80°C.

### Real-time quaking induced conversion (RT-QuIC) assay

Real-time quaking induced conversion (RT-QuIC), first described by Atarashi et al. [28], Wilham et al. [30], and Orru et al. [29], was used for the conversion of small quantities of prions present in the blood of TSE-infected animals. Positive assay controls and samples consisted of serial dilutions of a 10% homogenate of CWD or TME-infected brain (10^-3^-10^-9^) and NaPTA precipitated blood from infected animals (10^0^-10^-6^), respectively. Negative assay controls and samples were comprised of serial dilutions of a 10% homogenate of uninfected brain (10^-3^-10^-9^) and NaPTA precipitated blood from uninfected animals (10^0^-10^-6^), respectively. RT-QuIC reactions were set up in 96-well clear bottom optic plates (Nalgene Nunc) and consisted of 98 µl RT-QuIC Buffer (final concentrations of 1X PBS, 1 mM EDTA, 10 µM Thioflavin T (ThT), 100-200 mM NaCl buffer, and 0.1 mg/ml recombinant Syrian hamster PrP^C^ substrate) and 2 µl sample. Blood samples that were placed into the whole blood optimized RT-QuIC (WBO RT-QuIC) assay were 2 µl of serial dilutions made from concentrated material of 500 µl. Once reactions were set up in each well, plates were placed in a BMG Fluostar fluorescence plate reader with settings of 42°C for 60 hours with cycles consisting of 1 minute shake, 1 minute rest and ThT fluorescence measurements were taken every 15 minutes. Data were processed using Microsoft Excel (Microsoft Inc.) prior to graph production with Prism 6 (GraphPad Prism).

### Cervid immunohistochemistry

Samples were processed and analyzed as previously described by Nalls et al. [58]. In brief, fixed tissues were treated with formic acid, embedded in paraffin, cut, and placed on positively charged slides. Deparaffinized, rehydrated and PK digested (20 mg/ml) tissues underwent epitope retrieval and were probed with primary antibody BAR224 (Cayman Chemical) and secondary anti-mouse HRP labeled polymer (Dako) prior to counterstain and reading by light microscopy

### Hamster immunohistochemistry

Immunohistochemistry was performed to detect PrP^D^ as previously described [59]. In brief, deparaffinized, formic acid treated tissue sections were processed for antigen retrieval. Endogenous peroxidase and non-specific staining were blocked in H_2_O_2_ in methanol and normal horse serum. The sections were probed with monoclonal anti-PrP antibody 3F4 followed by secondary biotinylated horse anti-mouse immunoglobulin G conjugate prior to detection with ABC solution (Elite kit; Vector Laboratories). The sections were counterstained with hematoxylin and read by light microscopy 

### Western blotting

Western blotting performed as previously described [62] with the following modifications: tissue homogenates were mixed with proteinase K (PK) (Invitrogen) to a final concentration of 50 µg/ml and incubated at 37°C for 30 minutes, followed by incubation at 45°C for 10 minutes with constant agitation. Samples were size fractionated on a NuPAGE 10% Bis-Tris gel (Novex) in 1X MOPS buffer at 100 volts for 2.5 hours, transferred to a polyvinylidene fluoride (PVDF) membrane for 7 minutes using the Trans-blot Turbo transfer system (Biorad). Post-transfer, the PVDF membrane was loaded onto a wetted SNAP i.d. holder (Millipore) and placed in the SNAP i.d. vacuum filtration system (Millipore). The PVDF membrane was blocked for 10 minutes with Blocking Buffer (Blocker casein in TBS [Thermo Scientific] with 0.1% Tween 20), and incubated for 10 minutes with 0.2 µg/ml primary antibody BAR224 (Cayman Chemical) -HRP conjugated antibody. The membrane was washed with TBST and developed using ECL Plus enhanced chemiluminescence Western blotting detection reagents (Invitrogen) and imaged on a Luminescence image analyzer LAS 3000 (Fujifilm).

### Mouse titration bioassay

All animals were handled in strict accordance with guidelines for animal care and use provided by the United States Department of Agriculture (USDA), National Institutes of Health (NIH) and the Association for Assessment and Accreditation of Laboratory Animal Care International (AAALAC), and all animal work was approved by Colorado State University Institutional Animal Care and Use Committee (IACUC). Seven cohorts of TgCerPrP mice (n=9) were inoculated with 30 µl of a CWD-infected cervid brain homogenate intracranialy. Each cohort received a different concentration of inoculum ranging from 10% (w/v) to 0.00001% (w/v). Negative control mice were inoculated with sham material. Mice were subsequently observed and terminated upon onset of clinical disease. All mice were analyzed for the presence of PrP^D^ by Western blot and immunohistochemistry.

### Calculations

RT-QuIC assay sensitivity was determined by analyzing the number of replicates demonstrating positivity compared to the total number of replicates run (# of positive replicates/total replicates analyzed). Separate calculations were performed for animals with clinical disease status and animals with subclinical disease status.

PrP^D^ concentration in blood was determined by comparison of the time to positivity for whole blood and brain samples. 2 µl of a 1/100 dilution (blood seed=2x10^-2^ µl) of NaPTA treated whole blood was compared against a dilutional series of brain samples. A 10 % brain homogenate was used and serially diluted by 10-fold dilutions to 10^-8^. 2 µl of 10^-5^-10^-8^ dilutions were seeded into the RT-QuIC assay. Calculations for 10^-6^ dilution of brain in RT-QuIC are used as an example:0.1 g/ml diluted 10^-6^=(10^-10^ g/µl)(2 µl)=2x10^-10^ g=0.2 ng.

LD_50_ and SD_50_ were calculated using the Reed-Muench method [63].
